# Sequential PET/CT and pathological biomarker crosstalk predict response to PD-1 blockers alone or combined with sunitinib in propensity score-matched cohorts of cancer of unknown primary treatment

**DOI:** 10.3389/fonc.2023.1191611

**Published:** 2023-12-21

**Authors:** Youlong Wang, Qi Huang, Guanqing Zhong, Jun Lv, Qinzhi Guo, Yifei Ma, Xinjia Wang, Jiling Zeng

**Affiliations:** ^1^ Hainan Hospital of PLA General Hospital, Department of General Surgery, Haitang District, Sanya, China; ^2^ Department of Clinical Laboratory, State Key Laboratory of Oncology in South China, Collaborative Innovation Center for Cancer Medicine, Guangdong Key Laboratory of Nasopharyngeal Carcinoma Diagnosis and Therapy, Sun Yat-sen University Cancer Center, Guangzhou, China; ^3^ Department of Infectious Diseases, The First Affiliated Hospital of Zhengzhou University, Zhengzhou, China; ^4^ Pancreas Center of Guangdong Provincial People’s Hospital, Guangzhou, China; ^5^ Department of Spine Surgery, The Second Affiliated Hospital, Shantou University Medical College, Shantou, China; ^6^ Department of Nuclear Medicine, State Key Laboratory of Oncology in South China, Guangdong Provincial Clinical Research Center for Cancer, Sun Yat-sen University Center, Guangzhou, China

**Keywords:** cancer of unknow primary, PET/CT (18)F-FDG, sunitinb, EFGR, VEGFR

## Abstract

**Introduction:**

The efficacy of immune checkpoint inhibitors (ICIs), including toripalimab and pembrolizumab, has not been confirmed in the treatment of cancer of unknown primary (CUP), which has a very poor prognosis. Combined with anti-angiogenic therapies, ICIs are hypothesized to be effective in prolonging overall survival. The study aims to give evidence on the treatment effects of sunitinib combined with ICIs, find pathological biomarkers associated with changes in volumetric ^18^F FDG PET/CT parameters, and investigate inner associations among these markers associated with response on PET/CT.

**Methods:**

The study recruited patients receiving combined treatment (ICIs + sunitinib), compared the effects of combined treatment with those of separate treatment and age-matched negative controls, and analyzed propensity score-matched (PSM) pairs. Markers associated with survival were identified, and their inner associations were tested using structural equation modeling.

**Results:**

A total of 292 patients were enrolled in the final analysis, with 53 patients receiving combined treatment. Survival analysis demonstrated significantly prolonged survival in either combined or separate treatment, with the combined arm showing better response when PSM-paired using pre-treatment whole-body PET/CT parameters. The angiogenic markers KDR and VEGF mediate the PD-1 blockade impact on volumetric value changes in positive and negative manners.

**Conclusion:**

The anti-angiogenic agent sunitinib may potentiate PD-1 blockade by diminishing angiogenesis or its downstream effects. The combined separate treatment increased the survival of CUP patients, and the responses could be evaluated using volumetric PET/CT parameters.

## Introduction

Cancer of unknown primary (CUP) is defined as a heterogeneous group of malignancies with the primary site unable to be diagnosed using any current means (1). It has been recognized as an independent disease entity because of its distinct biological behavior, bio-aggressiveness, and pathological signatures (2). Its incidence is not uncommon, accounting for 2% to 5% of yearly incident cancers (3). Although our previous research found encouraging results of sunitinib therapy in CUP management, treatment strategies are still to be determined due to fluctuating therapeutic responses and difficulty of response evaluation (4).

Fortunately, over the last decades, immunotherapies have proved effective in prolonging survival in many solid or hematologic malignancies, shedding new light on the treatment of cancers that traditionally respond poorly to cytotoxic chemotherapy or targeted therapies (5, 6). Among them, immune checkpoint inhibitors (ICIs) by targeting PD-1 or PD-L1 modulate T-cell function and enhance cytotoxicity against tumor cells or deranged immune micro-environment (7). Although CUP has not been shown to respond to immunotherapies in piloting studies, combined drugs of anti-angiogenic agents and immune checkpoint inhibitors were demonstrated to have better effects in a landscape of multi-drug resistant solid tumors, but the regimens have not been studied in CUP patients (8, 9). We, therefore, aimed to test the efficacy of both therapies in CUP patients in either a combined or separate manner.

The second problem in diagnosing or treatment of CUP is biomarker profiling. Previous studies have attempted to find immune and pathological signatures in CUP patients, but they have not provided conclusive evidence on treatment response (8). Indeed, due to the complexity of the host immune system and its interplay with the occult primary lesion, biomarkers cannot be as easy to identify as known primary cancers. Nevertheless, aberrant angiogenesis was one of the main reasons for immune suppression of the T-cell subgroup, and therefore, this work seeks to identify penitential biomarkers associated with treatment response (5).

Different from known primary, the metastatic lesions are usually multiple, and the primary is occult, calling for a novel approach to evaluate treatment response (6). In the prior study of sunitinib therapy of CUP, the efficacy of volumetric bio-signatures of sequential PET/CT scans in drug response prediction has been shown to be independently associated with survival (10). This non-invasive method of evaluating tumor glycolysis combines the tumor volume and metabolic rate and thus has been shown to be superior to traditional measures (10, 11). In this study, to avoid unbalanced potential selection bias, propensity score-matched analyses were applied in comparison to treatment arms (12). Then, potential pathological biomarkers indicating response were analyzed. Finally, the interplay of the biomarkers was investigated using structure equation modeling to identify the indirect effects of biomarkers.

## Methods

### Patients

The open-labeled study recruited patients diagnosed with cancer of unknown primary who received treatment of sunitinib and immune checkpoint inhibitors at Sun Yat-sen University Cancer Center, Guangzhou, Changzheng Hospital, and had panoramic medical imaging (Panmedic) at the Second Affiliated Hospital of Shantou University, from June 2015 to May 2021. Randomization was based on demographic data and baseline whole-body PET/CT values into combination treatment or separate treatment. Patients not receiving either therapy were included in the study as negative controls. Because the evaluation of the primary site was unavailable using Response Evaluation Criteria in Solid Tumors, the primary goal was to estimate the efficacy of either combined or separate treatment, which was demonstrated using survival prognosis and changes in whole-body PET/CT metabolic signature, and the association between value changes on PET/CT and survival was analyzed. The inclusion criteria, PET/CT imaging, and immunohistochemistry method have been illustrated elsewhere (10, 13) (**Supplementary Materials**). The study was approved by the institutional review boards and was in accordance with the provisions of the Declaration of Helsinki. All patients provided written and/or oral consent to participation before the study commenced.

The dosage of sunitinib was 50 mg/day given in 6-week cycles, including 4 weeks on treatment followed by 2 weeks off treatment (Schedule 4/2), and dosage was reduced to 37.5 mg/day and subsequently to 25 mg/day on occasions of over grade 3 toxicity. Patients received toripalimab 3 mg/kg once every 2 weeks by intravenous infusion, and the dose was reduced to 2.5 mg/kg in occasions of unbearable toxicity. For the purpose of the study, the intention-to-treat manner was adopted in the subsequent analysis. The dosage of pembrolizumab was 200 mg every 3 weeks, and the dose was reduced to 130 to 180 mg in occasions of unbearable toxicity, which was defined as any toxicity of greater than grade III or any patient-reported toxicity to stop ICI treatment.

### Propensity score-matched analysis

The demographic variables were acquired from the medical records, and the overall condition of the patients was assessed using the Eastern Cooperative Oncology Group Performance Score (ECOG-PS). As there may have been a potential difference in variables not included in the study, patients in each treatment arm were matched by propensity score to reach a 1:1 paired comparison in order to minimize selection bias and confounding variables. Propensity score-matched analysis was carried out by means of a multivariate conditional logistic regression model with a caliper width of 0.05 (14). Factors included in the regression model included demographics, chemotherapy involved, and baseline metabolic activity on whole-body FDG PET/CT scans associated with tumor aggressiveness, including high standard uptake value (HSUV), whole-body metabolic tumor volume (WMTV), and whole-body total lesion glycolysis (WTLG).

### Statistics and data assessment

First, the unmatched survival curve of combined or separate treatment was calculated and plotted using the Kaplan–Meier method. Log-rank test was used to test the difference. Second, the propensity score was calculated in each treatment arm to achieve a matched analysis for all treatment arms. Paired Student’s t-test was applied to test differences in continuous variables, and the chi-square test was used to test categorical differences. In each treatment arm, univariate and multivariate survival analyses were applied to find independent risk variables associated with survival by means of Cox proportional hazards models. Finally, structural equation modeling (SEM) was performed to examine the direct or indirect effects of immunohistochemistry (IHC) markers on the value fluctuation of PET/CT metabolic biomarkers and survival. Only markers significant in the survival analysis would enter the model to test their significance and regression weights. Pearson’s correlation was considered to adjust regression weights if there was more than one variable at the beginning of SEM. The survival analysis was performed on SPSS (Chicago, IL, USA; version 24.0), and SEM was performed on Amos (Chicago, IL, USA; version 24.0).

## Results

### Baseline characteristics

A total of 299 patients were included in the study at baseline, of whom four patients failed to undergo a second PET/CT scan after treatment discontinuation, and three patients refused to provide information on PET/CT scans. Therefore, a total of 292 patients were enrolled finally (135 men and 157 women), including 43 patients receiving ICIs of toripalimab or pembrolizumab only, 57 patients receiving sunitinib therapy only, 53 patients receiving combined therapy, and 139 patients receiving neither (age-matched negative control). The mean and standard deviation (SD) values of baseline HSUV, WMTV, and WTLG were 18.34 ± 4.57, 56.97 ± 23.70, and 301.03 ± 77.55, respectively. A total of 108 patients were rated using ECOG-PS as 3 and 4, and 184 patients were rated as 1 and 2. The baseline information of all patients and each treatment arm is shown in **Supplementary Table 1**. The Kaplan–Meier curve demonstration of unmatched survival information is shown in **Supplementary Figure 1**.

### Propensity score-matched comparison

**Figure 1:**
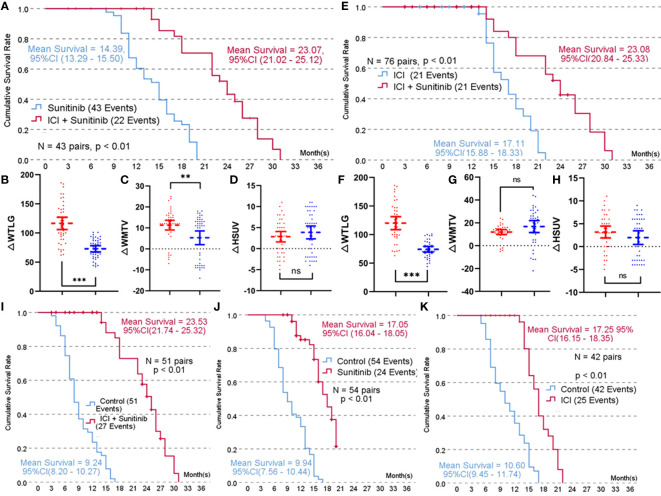
Propensity score-matched 1:1 comparison of each treatment arm. **(A)**, Kaplan-Meier survival curve of combined treatment (toripalimab or pembrolizumab + sunitinib) versus sunitinib treatment; **(B)**, Comparison result of improvement in whole-body total lesion glycolysis (△WTLG) in combined treatment versus sunitinib treatment; **(C)**, Comparison result of improvement in whole-body metabolic tumor volume (△WMTV) in combined treatment versus sunitinib treatment; **(D)**, Comparison result of improvement in highest standard uptake value (△HSUV) in combined treatment and sunitinib treatment; **(E)**, Kaplan-Meier survival curve of combined treatment versus immune checkpoint inhibitors (toripalimabor pembrolizumab) treatment; **(F)**, Comparison result of improvement in whole-body total lesion glycolysis (△WTLG) in combined treatment versus immune checkpoint inhibitors; **(G)**, Comparison result of improvement in whole-body metabolic tumor volume (△WMTV) in combined treatment versus immune checkpoint inhibitors; **(H)**, Comparison result of improvement in highest standard uptake value (△HSUV) in combined treatment versus immune checkpoint inhibitors; **(I–K)**, Comparison result of the mean estimated survival time among combined therapy, sunitinib therapy, and ICI therapy.

A propensity score-matched comparison was carried out in five paired groups to balance the baseline characteristics shown in **Table 1**. A total of 43 pairs were matched in combined therapy versus sunitinib therapy (mean score = 0.49 ± 0.12), and baseline characteristics comparison is shown in **Table 1**. The mean estimated survival time of the combined group was 23.07 months, with 95 confidence intervals (CIs) of 21.02–25.12, which was significantly longer than that of patients receiving sunitinib alone (**Figure 1A**). At the end of the follow-up PET/CT scan, both therapy arms demonstrated significant improvement in WTLG, WMTV, and HSUV compared with baseline parameters (p < 0.01 for all parameters, see **Table 1**). Changes (Δ) in WMTV and WTLG were significantly different between the combined therapy group and the sunitinib group (**Figures 1B, C**), but there was no significant difference in ΔHSUV between the two arms (**Figure 1D**).

**Table 1:** Propensity score-matched comparison results of combined therapy and separate therapies.

	ICI + sunitinib group *vs.* sunitinib group (N = 43 pairs)			ICI + sunitinib group *vs.* ICI group (N = 38 pairs)		
Factor	ICI + sunitinib	Sunitinib	p	ICI + sunitinib	ICI	p
Baseline variables						
Sex, male/female	16/27	19/24	0.51	17/21	18/20	0.82
Age, mean (SD)	56.93 (14.14)	57.77 (12.23)	0.77	56.89 (13.77)	56.84 (15.96)	0.99
Pathology type, SCC/adenoCA/UD	13/17/13	10/18/15	0.76	13/15/10	12/13/13	0.75
Chemotherapy, paclitaxel/pt/combined	18/12/13	14/17/12	0.50	11/10/17	11/15/12	0.39
ECOG-PS, 4/3/2/1	3/6/15/19	2/7/17/17	0.92	4/7/17/10	7/10/13/8	0.55
WTLG, mean (SD)	293.90 (72.34)	302.95 (71.91)	0.56	305.09 (74.05)	312.63 (70.86)	0.65
WMTV, mean (SD)	56.40 (23.94)	55.16 (22.05)	0.81	62.00 (25.35)	62.37 (24.84)	0.95
HSUV, mean (SD)	17.28 (4.67)	17.60 (4.55)	0.74	18.32 (4.61)	18.84 (4.65)	0.62
Follow-up PET/CT parameters						
WTLG, mean (SD)	177.34 (77.41)	230.02 (74.86)	<0.01	185.09 (77.87)	238.32 (68.77)	<0.01
ΔWTLG	116.56 (34.45)	72.93 (17.12)	<0.01	120.00 (34.53)	74.32 (15.25)	<0.01
WMTV, mean (SD)	45.07 (22.47)	49.86 (21.97)	0.32	50.05 (24.29)	45.58 (20.56)	0.39
ΔWMTV	11.33 (7.49)	5.30 (10.66)	<0.01	11.95 (6.53)	16.79 (15.96)	0.08
HSUV, mean (SD)	14.44 (3.86)	13.74 (4.01)	0.41	15.13 (3.64)	16.87 (4.03)	0.05
ΔHSUV	2.84 (4.02)	3.86 (4.98)	0.30	3.18 (3.98)	1.97 (4.58)	0.21

SD, standard deviation; ICI, immune checkpoint inhibitor; ECOG-PS, Eastern Cooperative Oncology Group Performance Score; Δ, improvement; WTLG, whole-body total lesion glycolysis; WMTV, whole-body metabolic tumor volume; HSUV, highest standardized uptake value.

A total of 38 pairs were matched in combined therapy versus ICI therapy (mean propensity score = 0.53 ± 0.12), and baseline characteristics comparison is shown in **Supplementary Table 3**. The mean estimated survival time of the combined group was significantly longer than that of patients receiving ICI alone (**Figure 1E**). At the end of the follow-up PET/CT scan, both therapy arms demonstrated significant improvement in WTLG, WMTV, and HSUV compared with baseline parameters (p < 0.01 for all parameters, **Supplementary Table 2**). ΔWTLG was significantly different between the combined therapy group and ICI group (**Figure 1F**), but there was no significant difference in ΔWMTV or ΔHSUV between the two arms (**Figures 1G, H**).

A total of 51, 54, and 42 pairs were matched in combined therapy, sunitinib therapy, and ICI therapy versus negative control. The mean propensity score of each match was 0.33 ± 0.12, 0.35 ± 0.14, and 0.33 ± 0.10, respectively. Baseline characteristics comparison is shown in **Supplementary Tables 2–4**. The mean estimated survival time of each treatment arm was significantly longer than that of the control group (**Figures 1I–K**).

### Identification of prognostic biomarkers

Survival analysis using univariate and subsequent multivariate methods was carried out in each treatment arm to identify markers associated with survival. In the combined treatment, ΔWTLG (hazard ratio (HR) = 0.96, 95%CI = 0.92–0.99) was the only marker in PET/CT independently associated with longer survival (**Supplementary Table 5**). In all IHC markers, PD-L1 (HR = 0.18, 95%CI = 0.05–0.64, **Supplementary Figure 2A**) and KDR (HR = 0.37, 95%CI = 0.10–1.36, **Supplementary Figure 2C**) were independently associated with significantly longer survival time. VEGF was found to be significantly associated with decreased survival prognosis (HR = 0.93, 95%CI = 0.29–3.02, **Supplementary Figure 2B**). Two IHC factors, however, were found to be associated with longer survival but lost significance in multivariate analysis (**Supplementary Figure 2D, E**), in which higher microvascular density was found negatively associated with survival and PDGFR was positively associated with survival.

In the sunitinib treatment arm, ΔWTLG was the only PET/CT biomarker associated with longer survival (HR = 0.98, 95%CI = 0.96–0.99). In all IHC markers, KDR was independently associated with significantly longer survival time (HR = 0.27, 95%CI = 0.12–0.59, **Supplementary Figure 2F**), and VEGF was independently associated with decreased survival time (HR = 3.63, 95%CI = 1.78–7.42, **Supplementary Figure 2G**, **Supplementary Table 6**).

In the ICI treatment arm, ΔWTLG was the only PET/CT biomarker associated with longer survival (HR = 0.96, 95%CI = 0.92–1.00). In all IHC markers, only PD-L1 was associated with longer survival (HR = 0.23, 95%CI = 0.07–0.78, **Supplementary Figure 2H**, **Supplementary Table 7**).

### PET/CT and pathological biomarkers correlate in structure equation modeling

Pathway analysis using structural equation modeling was carried out to unearth the inner association within the sensitive/resistant biomarkers and their direct or indirect impact on value changes of PET/CT volume-based biomarkers.

**Figure 2:**
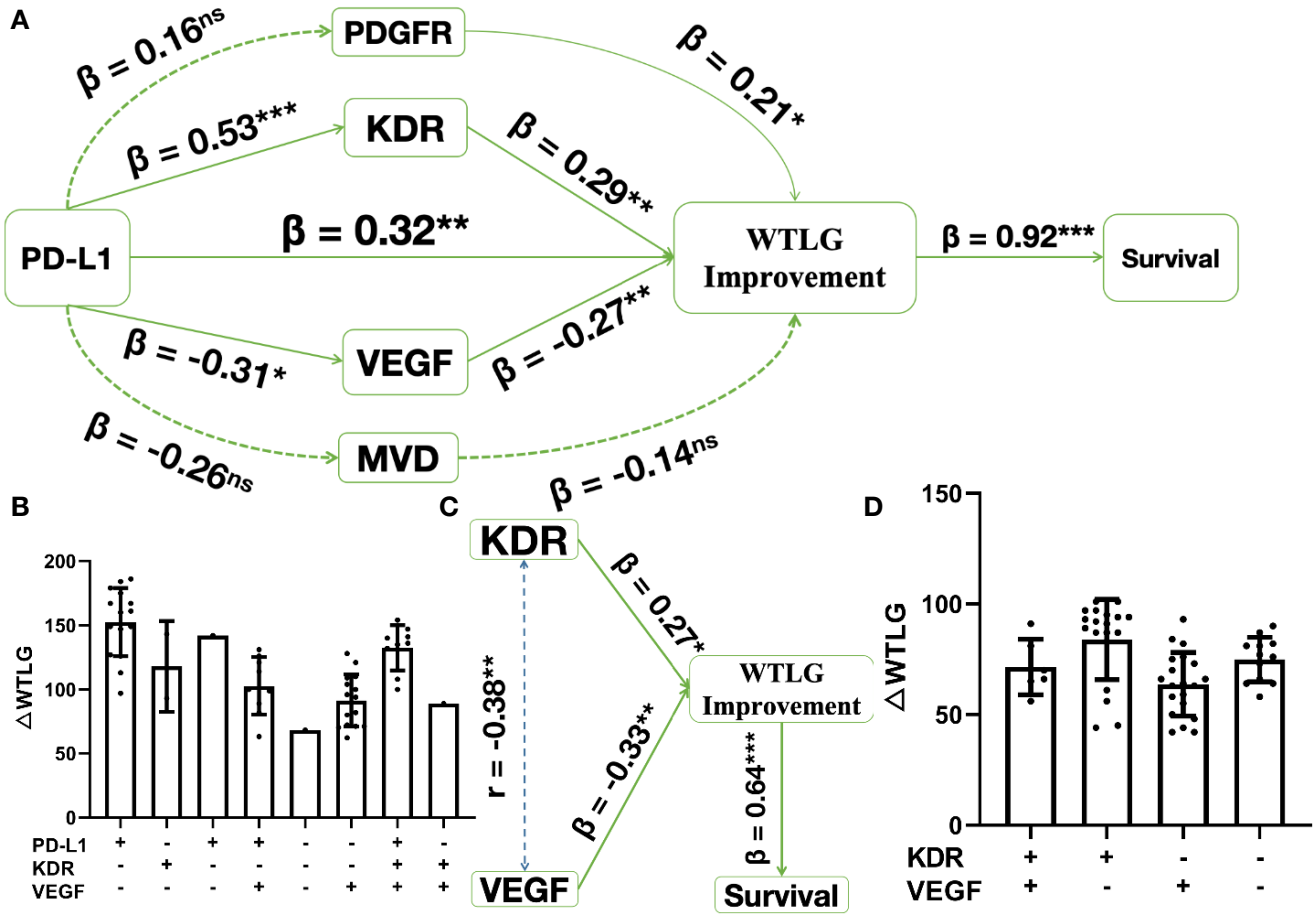
Structure equation modeling of biomarkers significant in survival analysis. * p < 0.05, **p < 0.01, ***p < 0.001, ns, insignificant. **(A)**, in combined treatment arm, pathway analysis shows PD-1 blockade has a direct impact on WTLG improvement (△WTLG) affecting survival. This impact is mediated by sunitinib treatment sensitivity, where KDR expression (β = 0.53 and 0.29) positively affects the impact and VEGF expression (β = -0.31 and -0.27) negatively affects the impacts; **(B)**, △WTLG in each subgroups of biomarker expression in combined treatment arm; **(C)**, in sunitinib treatment arm, both KDR and VEGF expression have direct impact on △WTLG affecting survival and there is weak correlation (Pearson r = 0.38) between the two biomarkers; **(D)**, △WTLG in each subgroups of biomarker expression in sunitinib treatment arm.

In the combined treatment arm (sunitinib combined with toripalimab or pembrolizumab), there was a direct impact of PD-1 blockade on ΔWTLG affecting survival, in which the regression coefficient for PD-L1 expression (β) was 0.32 on the impact of ΔWTLG (p < 0.01). Since there were four biomarkers of sunitinib therapy significant in the univariate survival analysis, indirect mediating effects were tested for these biomarkers in the pathway between PD-L1 and ΔWTLG. The final result is shown in **Figure 2A**. The impact of PD-1 blockade on ΔWTLG was positively mediated by KDR expression (β = 0.53 and β = 0.29, p < 0.01 for both) and by VEGF expression (β = −0.31 and β = −0.27, p < 0.05 and p < 0.01, respectively). There were two variables not significant in the pathway: PDGFR expression and microvascular density (MVD). The direct impact of ΔWTLG on survival was significant (β = 0.92, p < 0.01). Levels of ΔWTLG in each IHC expression subgroup are shown in **Figure 2B**.

In the sunitinib treatment arm, after adjustment by correlation analysis of KDR and VEGF expression (Pearson’s r = 0.38, p < 0.01), there was a direct impact of KDR expression on ΔWTLG (β = 0.27, p < 0.05), and there was also the direct impact of VEGF on ΔWTLG (β = −0.33, p < 0.01). The direct impact of ΔWTLG on survival was significant (β = 0.64, p < 0.01, **Figure 2C**). Levels of ΔWTLG in each IHC expression subgroup are shown in **Figure 2D**.

## Discussion

This work evaluated the efficacy of PD-1 inhibitors, including toripalimab and pembrolizumab, and sunitinib regimens in the treatment of CUP, analyzed the response-predicting role of sequential volume-based PET/CT scans, and investigated the inner associations of resistant or sensitive biomarkers. First, propensity score-matched cohorts demonstrated the survival prognosis of each treatment arm; second, multivariate analysis showed ΔWTLG to be the independent predictor of drug response and identified pathological markers of each treatment arm; finally, structure equation modeling analyzed the way anti-angiogenesis therapy assisted immune checkpoint blockade to achieve decreased tumor glycolysis in whole-body PET/CT scans. This study was the first to suggest the response of either combined or independent therapeutic efficacy of CUP.

Patients presenting with CUP may have their primary lesions concealed at the beginning or some point of the preclinical disease course for unknown reasons, and the occult primary site presents as an obstacle for precise diagnosis and subsequent management (15). Regardless of the pathogenesis of CUP or grouping methods into genetic subtypes, angiogenesis was aberrant and accelerated in many solid tumors, including CUP, in terms of the basic mechanism behind treatment regimens (16). Unleashed angiogenesis is one of the reasons for nourishing metastatic or primary tumors, and targeting angiogenesis is one of the main strategies in solid tumor treatment (16). Sunitinib, a multi-target receptor tyrosine kinase inhibitor, proved effective in metastatic renal cell carcinomas or gastric stromal cancers (13). The drug proved effective in CUP treatment in our previous work and was reevaluated in the present study, both of which identified VEGFR as the sensitive treatment biomarker and thus supported the anti-angiogenic effect of sunitinib (13, 17). A few other studies also illustrated the beneficial role of sunitinib in tumor immune surveillance combined with PD-L1 inhibitors (18–20).

Notably, since the treatment response of CUP can be difficult to evaluate with traditional measures as known primary tumors in Response Evaluation Criteria in Solid Tumors (RECIST) criteria, whole-body scans using PET/CT would be reasonably more actionable in clinical settings in evaluating prognosis or treatment response in CUP management (21). We evaluated the prognostic value of volumetric markers in the control group and found that the WTLG was the only marker associated with survival. Sequential PET/CT corroborated this result by demonstrating ΔWTLG as the only response predictor in the combined or independent therapies. Glycolysis bears more tumor information, as it is the product of tumor SUV and metabolic tumor volumes, and previous research using sequential PET/CT as prognostic markers has demonstrated the response-predicting role of glycolysis (10, 22). Some reports have given solid recommendations that WTLG should be applied in clinical settings as a standard measure of drug response (10, 23, 24). Our previous PANMEDIC report on CUP treatment demonstrated that whole-body glycolysis had more sensitivity and specificity in predicting survival in sunitinib treatment.

As traditional target therapies need appropriate biomarkers or sensitive genes to take clinical effect in certain malignancies, CUPs, being a heterogeneous group of cancers, may be immune to such therapies because concealed primary lesions may have blunted targets due to complex interplay of differential genes, and this also makes vigorous gene testing inapplicable to widespread relevance (25). In the last decade, however, immunotherapies, as represented by ICIs, bypass the genetic targeting in many solid cancers altogether (3, 26). The ICIs aim to rejuvenate exhausted host cytotoxic T cells to exert a potent effect on cancer cells, enabling efficient control of a landscape of solid or hematological malignancies. The effective treatment of CUP in the present study by pembrolizumab or toripalimab alone demonstrates that the immune checkpoint blockade may be effective in reducing progression, thus prolonging patient survival (27). Future randomized controlled trials are encouraged to give more conclusive evidence on CUP treatment.

Despite the fact that ICIs have significantly revolutionized cancer therapies, up to 60% of patients failed to have an adequate response by literature (28). Biomarkers associated with ICI response are difficult to identify, probably because the host immune system is too complex to be represented by independent biomarkers (28). Nevertheless, among the many resistant biomarkers, angiogenesis markers were also found to have crosstalk with T-cell immune function and survival, which has been reported to affect ICI therapy response in previous studies (29).

VEGF, being the “king” of angiogenesis, was found in the study to hinder anti-PD-1 therapeutic effects in the combined therapy group, where the structural equation modeling demonstrated that the VEGF expression levels had a negative impact on the PD-1 blockade response. KDR (VEGFR-2) expression level was found to positively mediate the effect of PD-1 blockade. However, both markers were not significantly associated with treatment response in separate treatment groups, suggesting that sunitinib therapy may diminish the effect of angiogenesis, thus potentiating immune blockade in combination treatment. Also, the indirect pathway by KDR bears more regression coefficients than the direct pathway (β = 0.53 versus β = 0.32), suggesting that the combined treatment needs higher KDR expression than PD-L1 expression to have an impact on WTLG improvement. Higher levels of KDR expression permitted higher anti-angiogenic effects, and thus, PD-1 blockade worked better, and subsequent WTLG improvement was higher. Indeed, the response-predicting results of both biomarkers of angiogenesis have been validated using the multivariate survival analysis and thus support previous data on the combined treatment of solid tumors that anti-angiogenesis may have a synergistic or permissive effect on PD-1 inhibition (30).

Interestingly, MVD was found to be insignificant in the mediating effects of PD-1 blockade, although, in preclinical settings, endothelial cells mediate decreased cytotoxic T lymphocyte (CTL) infiltration or increased T-cell apoptosis. Studies suggested that microvascular disorganization may not be the main reason for deranged CTL infiltration, and VEGF-associated downstream factors may play more important roles (30). However, the fact that VEGF instead of MVD mediates PD-1 blockade resistance in this study may need further investigation to clarify the mechanism.

This work bears limitations. Although matched comparison by propensity score was performed to determine the survival difference, the sample size is relatively small in each treatment arm, which calls for larger-scale research to be carried out in the future. Also, the research on biomarkers has not been extensive enough to involve genetic signatures, and therefore, future research can evolve into sequencing analysis on the basis of immunohistochemical markers, which could further unravel the inner workings of biomarkers behind combined therapy.

## Data availability statement

The original contributions presented in the study are included in the article/**Supplementary Material**. Further inquiries can be directed to the corresponding author.

## Ethics statement

The studies involving humans were approved by The second affiliated hospital of shantou university. The studies were conducted in accordance with the local legislation and institutional requirements. The participants provided their written informed consent to participate in this study.

## Author contributions

YW, QH,GZ, JL,QG, YM, XW, JZ contributed to the design and implementation of the research, to the analysis of the results and to the writing of the manuscript. All authors contributed to the article and approved the submitted version.
