# Corrigendum: Sequential PET/CT and pathological biomarker crosstalk predict response to PD-1 blockers alone or combined with sunitinib in propensity score-matched cohorts of cancer of unknown primary treatment

**DOI:** 10.3389/fonc.2024.1376408

**Published:** 2024-02-27

**Authors:** Youlong Wang, Qi Huang, Guanqing Zhong, Jun Lv, Qinzhi Guo, Yifei Ma, Xinjia Wang, Jiling Zeng

**Affiliations:** ^1^ Hainan Hospital of PLA General Hospital, Department of General Surgery, Haitang District, Sanya, China; ^2^ Department of Clinical Laboratory, State Key Laboratory of Oncology in South China, Collaborative Innovation Center for Cancer Medicine, Guangdong Key Laboratory of Nasopharyngeal Carcinoma Diagnosis and Therapy, Sun Yat-sen University Cancer Center, Guangzhou, China; ^3^ Department of Infectious Diseases, The First Affiliated Hospital of Zhengzhou University, Zhengzhou, China; ^4^ Pancreas Center of Guangdong Provincial People’s Hospital, Guangzhou, China; ^5^ Department of Spine Surgery, The Second Affiliated Hospital, Shantou University Medical College, Shantou, China; ^6^ Department of Nuclear Medicine, State Key Laboratory of Oncology in South China, Guangdong Provincial Clinical Research Center for Cancer, Sun Yat-sen University Center, Guangzhou, China

**Keywords:** cancer of unknow primary, PET/CT (18)F-FDG, sunitinb, EFGR, VEGFR

In the published article, there was an error in affiliation 4. The affiliation previously stated “^4^Pancreas Center of Guangdong Provincial People’s Hospital, Guangzhou, China”, the corrected affiliation is: “^4^Pancreas Center of Guangdong Provincial People’s Hospital, Guangzhou, China”.

In the published article, there was an error in affiliation 6. The previous affiliation stated “^6^Department of Nuclear Medicine, Sun Yat-sen University Cancer Center, Guangzhou, China”, the corrected affiliation is: “^6^Department of Nuclear Medicine, State Key Laboratory of Oncology in South China, Guangdong Provincial Clinical Research Center for Cancer, Sun Yat-sen University Center, Guangzhou, China”.

In the published article, an author name was incorrectly written as Wang Youlong. The correct spelling is Youlong Wang.

In the published article, an author name was incorrectly written as Guangqing Zhong. The correct spelling is Guanqing Zhong.

In the published article, the author contributions section was incorrectly written as “WY, QH, GZ, JL, QG, YM, XW, JZ contributed to the design and implementation of the research, to the analysis of the results and to the writing of the manuscript. All authors contributed to the article and approved the submitted version”. The corrected author contributions is “YW, QH, GZ, JL, QG, YM, XW, JZ contributed to the design and implementation of the research, to the analysis of the results and to the writing of the manuscript. All authors contributed to the article and approved the submitted version”.

In the published article, the funding was mistakenly not included in the publication. The missing funding appears below:

“This work was supported by the Science and Technology Special Fund of Guangdong Province of China [STKJ2023002]”.

The authors apologize for these errors and state that this does not change the scientific conclusions of the article in any way. The original article has been updated.

