# Supplementary material for: Sequential PET/CT and pathological biomarker crosstalk predict response to PD-1 blockers alone or combined with sunitinib in propensity score-matched cohorts of cancer of unknown primary treatment
Source: Front Oncol. 2023 Dec 21;13:1191611. doi: 10.3389/fonc.2023.1191611 (PMC10777842; doi:10.3389/fonc.2023.1191611)
Supplement: Supplementary file 1 [file Table_1.docx]

| Supplementary Table 1. Baseline Characteristics of Enrolled Patients | | | | | |
| --- | --- | --- | --- | --- | --- |
| **Factor** | **Total** | **ICI + Sunitinib** | **Sunitinib alone** | **ICI alone** | **Negative Control** |
| Sex, Male / Female | 135 / 157 | 23 / 30 | 26 /31 | 22 / 21 | 64 / 75 |
| Age, Mean (SD) | 59.14 (14.29) | 58.45 (13.95) | 55.61 (12.80) | 58.56 (16.48) | 61.04 (14.11) |
| Pathology type, SCC / adenoCA / UD | 91 / 102 / 99 | 14 / 19 / 20 | 19 / 22 / 16 | 16 / 13 / 14 | 42 / 48 / 49 |
| Chemotherapy, paclitaxel / pt /combined | 100 / 105 / 87 | 18 / 14 / 21 | 22 / 22 13 | 11 / 17 / 15 | 49 / 52 / 38 |
| ECOG-PS,  4 / 3 / 2 / 1 | 39 / 69 / 96 / 88 | 3 / 11 / 17 / 22 | 3 / 11 / 23 / 20 | 5 / 8 / 19 / 11 | 28 / 39 / 37 / 35 |
| WTLG, Mean (SD) | 301.03 (77.55) | 297.60 (75.22) | 306.09 (73.15) | 309.79 (72.37) | 297.56 (82.05) |
| WMTV, Mean (SD) | 56.97 (23.70) | 58.66 (24.44) | 54.68 (21.94) | 62.09 (24.10) | 55.68 (23.96) |
| HSUV, Mean (SD) | 18.34 (4.57) | 17.08 (4.55) | 18.65 (4.56) | 19.58 (5.10) | 18.30 (4.33) |
| ICI, Immune Checkpoint Inhibitors; ECOG-PS, Eastern Cooperative Oncology Group Performance Score；WTLG, Whole-body Total Lesion Glycolysis; WMTV, Whole-body Metabolic Tumor Volume; HSUV, Highest Standardized Uptake Value; pt, Platinum-based Chemotherapy; SCC, Squamous Cell Carcinoma; UD, Undifferentiated Tumor; adenoCA, adenocarcinoma. | | | | | |

| Supplementary Table 2. Propensity Score-matched Comparison Results of Combined Therapy and Control | | | |
| --- | --- | --- | --- |
| **Factor** | **ICI + Sunitinib** | **Control** | **p** |
| Baseline Characteristics | | | |
| Sex, Male / Female | 22 / 29 | 23 / 28 | 0.84 |
| Age, Mean (SD) | 59.14 (13.74) | 58.33(14.50) | 0.77 |
| Pathology type, SCC / adenoCA / UD | 13 / 18 / 20 | 17 / 14 / 20 | 0.60 |
| Chemotherapy, paclitaxel / pt /combined | 17 / 14 / 20 | 13 / 22 / 16 | 0.25 |
| ECOG-PS, 4 / 3 / 2 / 1 | 3 / 11 / 17 / 20 | 2 / 13 / 18 / 18 | 0.94 |
| WTLG, Mean (SD) | 298.41 (74.01) | 297.49 (81.93) | 0.95 |
| WMTV, Mean (SD) | 58.76 (24.74) | 61.25 (23.87) | 0.61 |
| HSUVmax, Mean (SD) | 17.27 (4.52) | 17.69 (4.53) | 0.65 |
| SD, standard deviation; ICI, immune checkpoint inhibitors; ECOG-PS, Eastern Cooperative Oncology Group Performance Score；△, Improvement; WTLG, whole-body total lesion glycolysis; WMTV, whole-body metabolic tumor volume; HSUV, highest standardized uptake value. | | | |

| Supplementary Table 3. Propensity Score-matched Comparison Results of Sunitinib Therapy and Control | | | |
| --- | --- | --- | --- |
| **Factor** | **Sunitinib** | **Control** | **p** |
| Baseline Characteristics | | | |
| Sex, Male / Female | 24 / 30 | 23 / 31 | 0.85 |
| Age, Mean (SD) | 56.22 (12.87) | 54.46 (13.20) | 0.48 |
| Pathology type, SCC / adenoCA / UD | 18 / 20 / 16 | 21 / 17 / 16 | 0.79 |
| Chemotherapy, paclitaxel / pt /combined | 19 / 22 / 13 | 19 / 23 /12 | 0.97 |
| ECOG-PS, 4 / 3 / 2 / 1 | 3 / 11 / 22 / 18 | 8 / 12 / 16 / 18 | 0.35 |
| WTLG, Mean (SD) | 304.04 (74.12) | 306.72 (74.04) | 0.85 |
| WMTV, Mean (SD) | 54.37 (21.86) | 53.50 (23.38) | 0.84 |
| HSUVmax, Mean (SD) | 18.78 (4.56) | 19.24 (4.57) | 0.60 |
| SD, standard deviation; ECOG-PS, Eastern Cooperative Oncology Group Performance Score；△, Improvement; WTLG, whole-body total lesion glycolysis; WMTV, whole-body metabolic tumor volume; HSUV, highest standardized uptake value. | | | |

| Supplementary Table 4. Propensity Score-matched Comparison Results of ICI Therapy and Control | | | |
| --- | --- | --- | --- |
| **Factor** | **ICI** | **Control** | **p** |
| Baseline Characteristics | | | |
| Sex, Male / Female | 21 / 21 | 20 / 22 | 0.83 |
| Age, Mean (SD) | 58.31(16.60) | 57.76(14.04) | 0.87 |
| Pathology type, SCC / adenoCA / UD | 15 / 13 / 14 | 16 / 13 / 13 | 0.97 |
| Chemotherapy, paclitaxel / pt /combined | 11 / 17 / 14 | 10 / 20 / 12 | 0.80 |
| ECOG-PS, 4 / 3 / 2 / 1 | 5 / 8 / 19 / 10 | 7 / 11 / 14 / 10 | 0.67 |
| WTLG, Mean (SD) | 311.05(72.77) | 316.71(78.81) | 0.73 |
| WMTV, Mean (SD) | 62.17(24.39) | 57.12(22.84) | 0.33 |
| HSUVmax, Mean (SD) | 19.40(5.03) | 19.62(4.72) | 0.84 |
| SD, standard deviation; ICI, immune checkpoint inhibitors; ECOG-PS, Eastern Cooperative Oncology Group Performance Score；△, Improvement; WTLG, whole-body total lesion glycolysis; WMTV, whole-body metabolic tumor volume; HSUV, highest standardized uptake value. | | | |

| Supplementary Table 5. Survival Analysis of PET/CT and Pathological Biomarkers in Combined Treatment Arm | | | | |
| --- | --- | --- | --- | --- |
| **Factor** | **Univariate** | **p** | **Multivariate** | **p** |
| Age | 0.98 (0.95 - 1.01) | 0.11 | — | — |
| Gender | 0.77 (0.35 - 1.73) | 0.53 | — | — |
| Chemotherapy | 1.10 (0.71 - 1.69) | 0.68 | — | — |
| ECOG-PS | 1.16 (0.76 - 1.77) | 0.50 | — | — |
| Pathology | 1.00 (0.62 - 1.63 ) | 0.99 | — | — |
| CAIX | 0.63 (2.52 - 1.58) | 0.33 | — | — |
| mTOR | 0.67 (0.29 - 1.56) | 0.36 | — | — |
| MVD + / - | 3.99 (1.44 - 11.07) | <0.01 | 2.32 (0.74 - 7.30) | 0.15 |
| PDGFR + / - | 0.20 (0.07 - 0.57) | <0.01 | 0.88 (0.25- 3.14) | 0.33 |
| PD-L1 + / - | 0.15 (0.05 - 0.40) | < 0.01 | 0.18 (0.05 - 0.64) | 0.01 |
| KDR | 0.13 (0.05 - 0.37) | < 0.01 | 0.37 (0.10 - 1.36) | 0.13 |
| VEGF | 2.72 (1.16 - 6.43) | 0.02 | 0.93 (0.29 - 3.02) | 0.90 |
| HSUV | 1.02 (0.93 - 1.11) | 0.67 | — | — |
| WMTV | 1.00 (0.99 - 1.02) | 0.83 | — | — |
| WTLG | 1.00 (0.99 - 1.00) | 0.25 | — | — |
| △HSUV | 0.94 (0.83 - 1.06) | 0.32 | — | — |
| △WMTV | 0.98 (0.92 - 1.04) | 0.57 | — | — |
| △WTLG | 0.94 (0.92 - 0.97) | < 0.01 | 0.96 (0.92 - 0.99) | 0.02 |
| ECOG-PS, Eastern Cooperative Oncology Group Performance Score；△, Improvement; WTLG, whole-body total lesion glycolysis; WMTV, whole-body metabolic tumor volume; HSUV, highest standardized uptake value. | | | | |

| Supplementary Table 6. Survival Analysis of PET/CT and Pathological Biomarkers in Sunitinib Treatment Arm | | | | |
| --- | --- | --- | --- | --- |
| **Factor** | **Univariate** | **p** | **Multivariate** | **p** |
| Age | 1.01 (0.99 - 1.03) | 0.43 | — | — |
| Gender | 0.93 (0.55 - 1.56) | 0.78 | — | — |
| Chemotherapy | 0.99 (0.70 - 1.39) | 0.94 | — | — |
| ECOG-PS | 0.81 (0.59 - 1.11) | 0.20 | — | — |
| Pathology | 0.92 (0.66 - 1.28) | 0.61 | — | — |
| CAIX | 1.48 (0.81 - 2.69) | 0.20 | — | — |
| mTOR | 0.97 (0.58 - 1.64) | 0.92 | — | — |
| MVD | 0.89 (0.50 - 1.56) | 0.68 | — | — |
| PDGFR | 0.58 (0.30 - 1.10) | 0.10 | — | — |
| PD-L1 | 0.80 (0.47 - 1.38) | 0.42 | — | — |
| KDR | 0.18 (0.09 - 0.38) | < 0.01 | 0.27 (0.12 - 0.59) | < 0.01 |
| VEGF | 5.89 (2.95 - 11.76) | < 0.01 | 3.63 (1.78 - 7.42) | < 0.01 |
| HSUV | 1.00 (0.94 - 1.06) | 0.93 | — | — |
| WMTV | 1.00 (0.99 - 1.01) | 0.92 | — | — |
| WTLG | 1.00 (1.00 - 1.01) | 0.25 | — | — |
| △HSUV | 0.99 (0.93 - 1.05) | 0.63 | — | — |
| △WMTV | 1.00 (0.97 - 1.02) | 0.82 | — | — |
| △WTLG | 0.98 (0.97 - 0.99) | < 0.01 | 0.98 (0.96 - 0.99) | 0.01 |
| ECOG-PS, Eastern Cooperative Oncology Group Performance Score；△, Improvement; WTLG, whole-body total lesion glycolysis; WMTV, whole-body metabolic tumor volume; HSUV, highest standardized uptake value. | | | | |

| Supplementary Table 7. Survival Analysis of PET/CT and Pathological Biomarkers in ICI Treatment Arm | | | | |
| --- | --- | --- | --- | --- |
| **Factor** | **Univariate** | **p** | **Multivariate** | **p** |
| Age | 0.96 (0.95 - 0.99) | 0.01 | 0.97 (0.94-1.00) | 0.06 |
| Gender | 0.83 (0.26 - 2.67) | 0.32 | — | — |
| Chemotherapy | 0.91 (0.54 - 1.52) | 0.72 | — | — |
| ECOG-PS | 0.92 (0.58 - 1.45) | 0.71 | — | — |
| Pathology | 1.01 (0.65 - 1.58) | 0.97 | — | — |
| CAIX | 1.12 (0.51 - 2.50) | 0.77 | — | — |
| mTOR | 0.59 (0.26 - 1.32) | 0.20 | — | — |
| MVD | 0.94 (0.42 -2.12) | 0.88 | — | — |
| PDGFR | 2.12 (0.93 - 4.82) | 0.07 | — | — |
| PD-L1 | 0.25 (0.08 - 0.77) | 0.02 | 0.23 (0.07 - 0.78) | 0.02 |
| KDR | 1.21 (0.55 - 2.66) | 0.64 | — | — |
| VEGF | 2.79 (0.98 - 7.90) | 0.05 | — | — |
| HSUV | 0.95 (0.88 - 1.03) | 0.19 |  |  |
| WMTV | 1.01 (0.97 - 1.03) | 0.26 |  |  |
| WTLG | 1.00 (0.99 - 1.00) | 0.34 | — | — |
| △HSUV | 1.00 (0.88 - 1.12) | 0.94 | — | — |
| △WMTV | 1.00 (0.98 - 1.02) | 0.18 | — | — |
| △WTLG | 0.96 (0.93 - 0.99) | 0.01 | 0.96 (0.92-1.00) | 0.03 |
| ECOG-PS, Eastern Cooperative Oncology Group Performance Score；△, Improvement; WTLG, whole-body total lesion glycolysis; WMTV, whole-body metabolic tumor volume; HSUV, highest standardized uptake value. | | | | |

| Supplementary Table 8. Survival Analysis of PET/CT and Pathological Biomarkers in Negative Control | | | | |
| --- | --- | --- | --- | --- |
| **Factor** | **Univariate** | **p** | **Multivariate** | **p** |
| Age | 1.00 (0.99 - 1.00) | 0.59 | — | — |
| Gender | 1.244 (0.89 - 1.74) | 0.20 | — | — |
| Chemotherapy | 0.81 (0.65 - 1.01) | 0.06 | — | — |
| ECOG-PS | 1.03 (0.88 - 1.20) | 0.74 | — | — |
| Pathology | 0.90 (0.74 - 1.10) | 0.31 | — | — |
| CAIX | 0.71 (0.50 - 1.01) | 0.06 | — | — |
| mTOR | 1.08 (0.76 - 1.55) | 0.66 | — | — |
| MVD | 1.21 (0.84 - 1.76) | 0.30 | — | — |
| PDGFR | 1.35 (0.96 - 1.91) | 0.08 | — | — |
| PD-L1 | 1.03 (0.74 - 1.45) | 0.86 | — | — |
| KDR | 0.96 (0.69 - 1.35) | 0.82 | — | — |
| VEGF | 1.32 (0.93 - 1.88) | 0.13 | — | — |
| HSUV | 0.98 (0.94 - 1.01) | 0.21 | — | — |
| WMTV | 1.00 (0.99 - 1.00) | 0.86 | — | — |
| WTLG | 0.99 (0.99 - 0.99) | < 0.01 | 0.99 (0.99 - 0.99) | < 0.01 |

| ECOG-PS, Eastern Cooperative Oncology Group Performance Score；△, Improvement; WTLG, whole-body total lesion glycolysis; WMTV, whole-body metabolic tumor volume; HSUV, highest standardized uptake value. |
| --- |
