# Supplementary material for: Sequential PET/CT and pathological biomarker crosstalk predict response to PD-1 blockers alone or combined with sunitinib in propensity score-matched cohorts of cancer of unknown primary treatment
Source: Front Oncol. 2023 Dec 21;13:1191611. doi: 10.3389/fonc.2023.1191611 (PMC10777842; doi:10.3389/fonc.2023.1191611)
Supplement: Supplementary file 2 [file DataSheet_1.docx]

**Figure legends**

**Supplementary Figure 1.** Unmatched survival analysis in each treatment arm.

**Figure 1.** Propensity score-matched 1:1 comparison of each treatment arm. 1A, Kaplan-Meier survival curve of combined treatment (toripalimab or pembrolizumab + sunitinib) versus sunitinib treatment; 1B, Comparison result of improvement in whole-body total lesion glycolysis (△WTLG) in combined treatment versus sunitinib treatment; 1C, Comparison result of improvement in whole-body metabolic tumor volume (△WMTV) in combined treatment versus sunitinib treatment; 1D, Comparison result of improvement in highest standard uptake value (△HSUV) in combined treatment and sunitinib treatment; 1E, Kaplan-Meier survival curve of combined treatment versus immune checkpoint inhibitors (toripalimabor pembrolizumab) treatment; 1F, Comparison result of improvement in whole-body total lesion glycolysis (△WTLG) in combined treatment versus immune checkpoint inhibitors; 1G, Comparison result of improvement in whole-body metabolic tumor volume (△WMTV) in combined treatment versus immune checkpoint inhibitors; 1H, Comparison result of improvement in highest standard uptake value (△HSUV) in combined treatment versus immune checkpoint inhibitors;

**Supplementary Figure 2.** Kaplan-Meier survival curve of pathological biomarkers in each treatment arm (Combined treatment and independent treatment). 2A, survival curve by PD-L1 expression in the combined treatment arm; 2B, survival curve by VEGF expression in the combined treatment arm; 3C, survival curve by KDR expression in the combined treatment arm; 2D, survival curve by PDGFR expression in the combined treatment arm; 2E, survival curve by microvascular density (MVD) in the combined treatment arm; 2F, survival curve by KDR expression in the sunitinib treatment arm; 2G, survival curve by VEGF expression in the sunitinib treatment arm; 2H, survival curve by PD-L1 expression in the immune checkpoint inhibitor arm;

**Figure 2.** Structure equation modeling of biomarkers significant in survival analysis. * p < 0.05, **p < 0.01, ***p < 0.001, ns, insignificant. 2A, in combined treatment arm, pathway analysis shows PD-1 blockade has a direct impact on WTLG improvement (△WTLG) affecting survival. This impact is mediated by sunitinib treatment sensitivity, where KDR expression (β = 0.53 and 0.29) positively affects the impact and VEGF expression (β = -0.31 and -0.27) negatively affects the impacts; 2B, △WTLG in each subgroups of biomarker expression in combined treatment arm; 2C, in sunitinib treatment arm, both KDR and VEGF expression have direct impact on △WTLG affecting survival and there is weak correlation (Pearson r = 0.38) between the two biomarkers; 2D, △WTLG in each subgroups of biomarker expression in sunitinib treatment arm.
