# Supplementary material for: Sequential PET/CT and pathological biomarker crosstalk predict response to PD-1 blockers alone or combined with sunitinib in propensity score-matched cohorts of cancer of unknown primary treatment
Source: Front Oncol. 2023 Dec 21;13:1191611. doi: 10.3389/fonc.2023.1191611 (PMC10777842; doi:10.3389/fonc.2023.1191611)
Supplement: Supplementary file 3 [file DataSheet_2.docx]

**Inclusion Criteria**

Patients must have had thorough clinical, endoscopic, and radiological investigations without any clue as to the site of primary lesion. Patients with one lymph node lesion were excluded (n = 4). By means of surgical biopsy, the pathological lesion of the visceral, bone, or lymph nodes was used to confirm the diagnosis of cancer of unknown primary (CUP).

**Immunohistochemistry**

The expression of potential sunitinib and ICI biomarkers were assessed by immunohistochemistry, including VEGF, VEGF receptor-2 (KDR), PDGFR, carbonic anhydrase IX (CAIX), mTOR, CD31. KDR was involved in analysis because of high vascularity as consulted with the pathologists, and CD31 was used as the scale to delineate microvascular density (MVD) according to Gundersen et al^1^. Microvessels were counted in five different fields with vascularity at 250 times of magnification. One CD31-positive vessel corresponded to one group of positive endothelial cells^2^.

Tumor PD-L1 protein expression was assessed with the use of an automated immunohistochemical assay kit (Dako) applying rabbit antihuman PD-L1 antibody (clone 28-8 and 22C3, Epitomics). PD-L1 + was confirmed when membrane staining of any intensity was observed at levels of 1% or higher, 5% or higher, and 10% or higher in a section including > 100 tumor cells that could be evaluated.

**PET/CT imaging and analysis**

18F-FDG-PET/CT was performed 0 – 20 days (median = 7 day) before therapy. The second scan was performed 12 to 19 days (median 10 days) after cycles 2 to 4 (week 12 or 24). if the patient discontinued the treatment, the PET/CT scan was performed after the last drug dose. The control cohort was scanned with PET/CT only at baseline to test the imaging signature sensitivity in association with survival.

Sixty minutes following injection of 370MBq of 18F-FDG after 5 to 9 hours of fasting, patients were scanned with PET/CT scanners (Biograph, Siemens, combined with CT). those with hyperglycemia were not scanned until the next time when the value of glucometers was within the normal range. The images were reconstructed with a maximization algorithm of standard ordered subset expectation. Patients in both medical centers were scanned with scanners of the same algorithm, procedures, and machine batch.

For imaging masking and parameter analysis, E-soft (4.0, Siemens Medical Solutions) was applied for each whole-body image. The threshold of 50% was applied in the delineation of tumor contours. The highest standard uptake value (HSUV) was defined as the highest SUV of all lesions in the whole-body imaging analysis. Whole-body metabolic tumor volume was defined as the summation of metabolic tumor volumes of all lesions on PET images, which has been reported as a quantitative measurement of tumor cells with high metabolic activity. To simultaneously assess the 18-F FDG uptake and tumor volume, the total lesion glycolysis (TLG) was calculated as the product of MTV and average SUV. Whole-body TLG (WTLG) was the summation of TLG of all lesions in the images. Changes (△) in WTLG, WMTV, and HSUV were defined as baseline parameters minus the follow-up parameters of each patient (△WTLG, △WMTV, and △HSUV).

1. Gundersen HJ, Bendtsen TF, Korbo L, et al. Some new, simple and efficient stereological methods and their use in pathological research and diagnosis. *APMIS : acta pathologica, microbiologica, et immunologica Scandinavica* 1988;96(5):379-94. doi: 10.1111/j.1699-0463.1988.tb05320.x

2. del Puerto-Nevado L, Rojo F, Zazo S, et al. Active angiogenesis in metastatic renal cell carcinoma predicts clinical benefit to sunitinib-based therapy. *British journal of cancer* 2014;110(11):2700-7. doi: 10.1038/bjc.2014.225
