# Supplementary figures and images for: Sequential PET/CT and pathological biomarker crosstalk predict response to PD-1 blockers alone or combined with sunitinib in propensity score-matched cohorts of cancer of unknown primary treatment
Source: Front Oncol. 2023 Dec 21;13:1191611. doi: 10.3389/fonc.2023.1191611 (PMC10777842; doi:10.3389/fonc.2023.1191611)

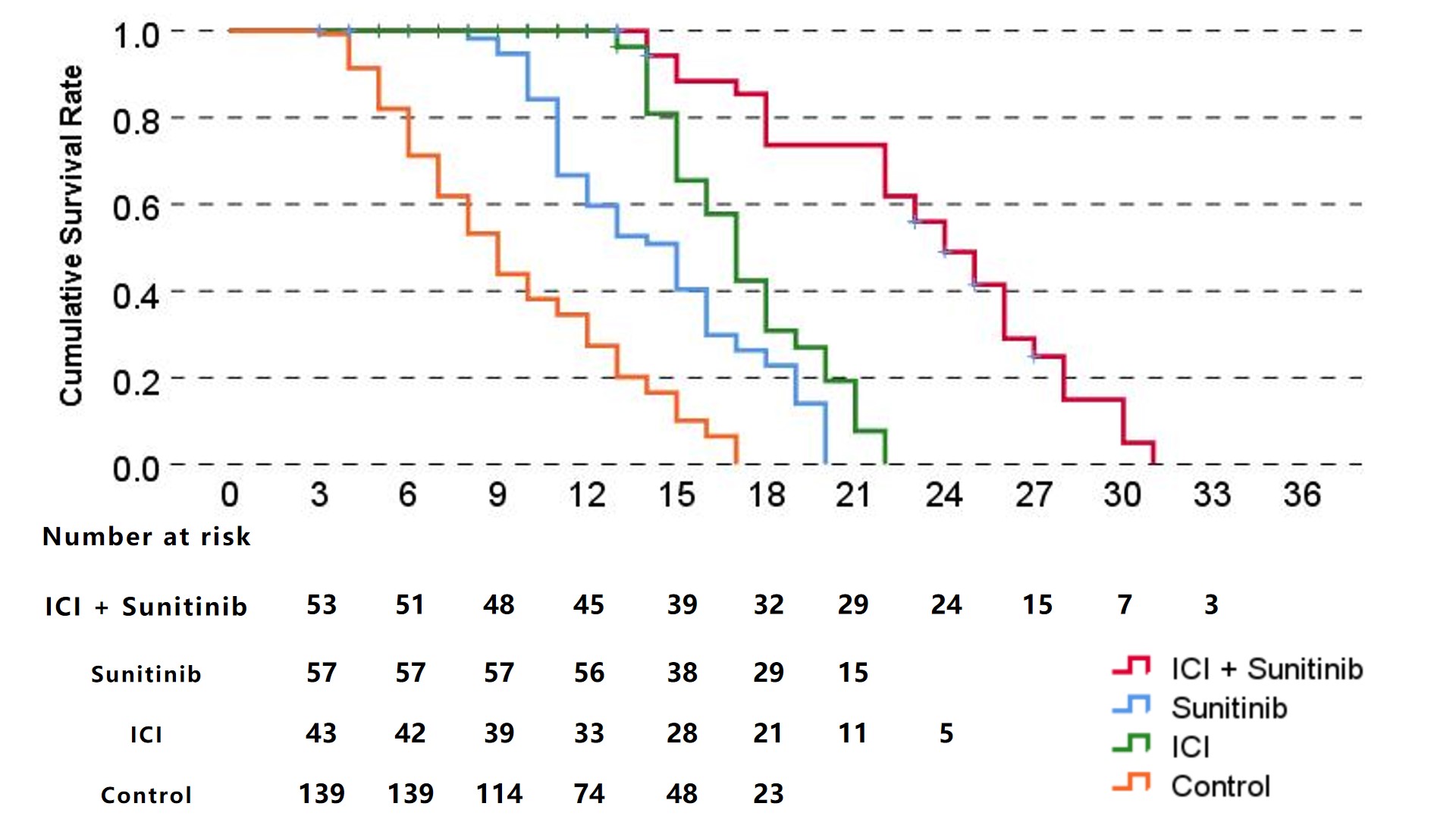

Supplement: Supplementary file 4 [file Image_1.jpeg]

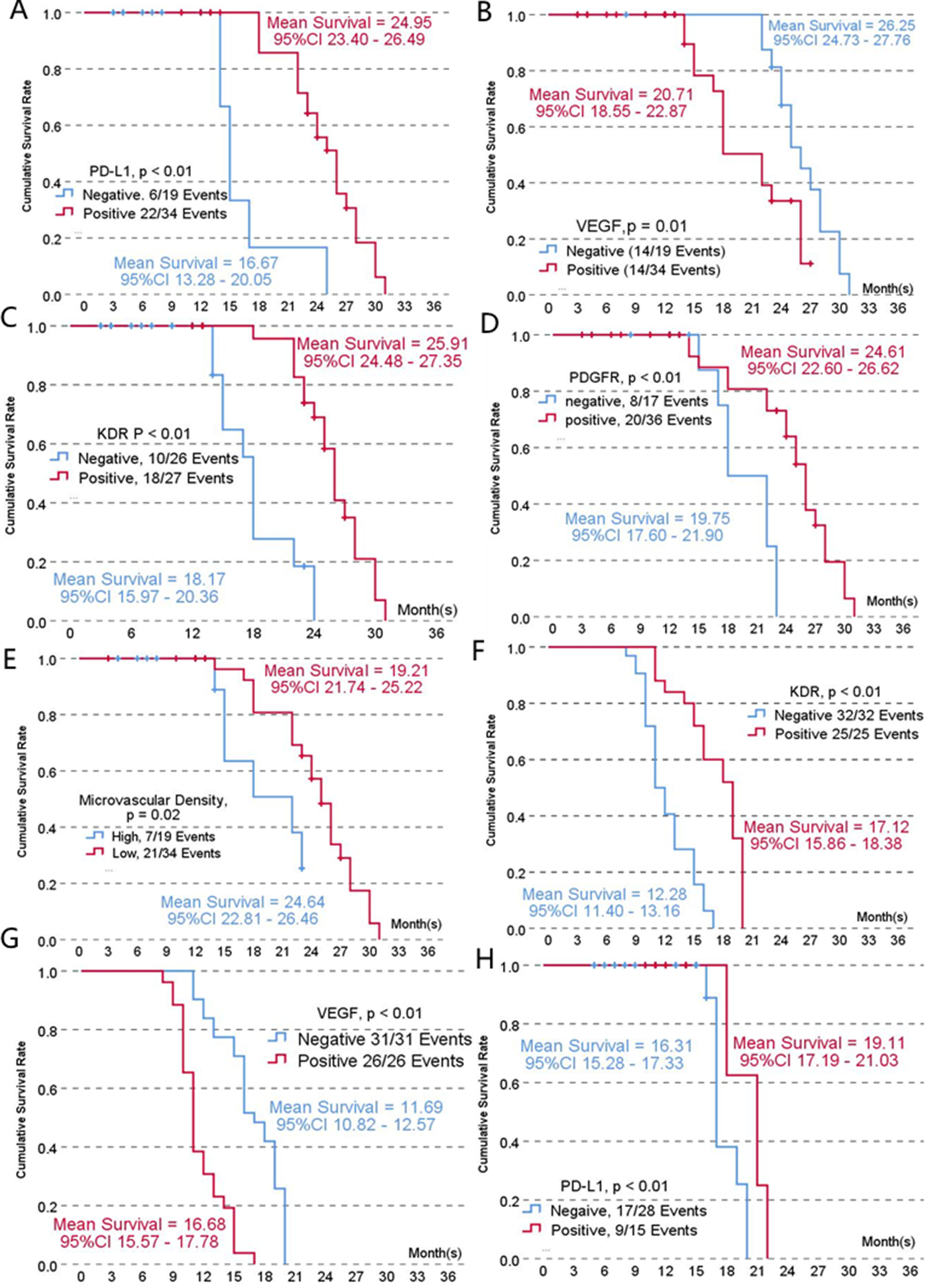

Supplement: Supplementary file 5 [file Image_2.jpeg]
